# On the verge between the scientific and the alternative: Swedish women’s claims about systemic side effects of the copper intrauterine device

**DOI:** 10.1177/09636625221107505

**Published:** 2022-07-28

**Authors:** Lena Gunnarsson, Maria Wemrell

**Affiliations:** Örebro University, Sweden; Lund University, Sweden

**Keywords:** copper intrauterine device, epistemic democracy, health activism, social media, women’s health

## Abstract

The article intervenes in discussions on lay knowledge production about health in the Internet era, through the case of a group of women claiming that their use of copper intrauterine devices has led to systemic side effects. Based on online group interviews and written essays, we examine how women embracing these knowledge claims navigate various sources of information, focusing on the role of scientificity in these epistemic negotiations. The women were found to be involved in an active, scientifically oriented process of knowledge formation, which we refer to as a *collective labour of scientific patchworking*. Meanwhile, due to a perceived lack of scientifically based expertise on their condition, the women reported having little choice but turn to resources with weaker scientific foothold. We argue that the tendency to portray these women’s claims as unscientific simplifies the nature of lay knowledge production, potentially deepening divides between medical authorities and the public.

The Internet has revolutionized people’s ways of seeking and sharing information and, consequently, their relationship to established epistemic authorities. Health is an arena where this transformation of the public’s relationship to established expertise has potentially noteworthy effects, and it has been suggested that ‘the contestation of expertise is perhaps nowhere more pronounced’ than in the field of health (Vuolanto et al., 2020: 508). In 1999, before the contemporary heyday of Internet technology and its permeation of every sphere of our lives, Michael Hardey (1999) envisioned that ‘the Internet forms the site of a new struggle over expertise in health that will transform the relationship between the health professions and their clients’ (p. 820). Subsequent research seems to confirm his prediction, showing that a great majority of Internet users have sought health-related information online, with social media as increasingly important platforms (Madathil et al., 2015).

Health-related information on the Internet stems from a variety of actors and can contradict information provided by healthcare institutions (Johnson et al., 2020). Hence, there is a considerable and widespread concern about misinformation and its consequences for individual and public health (Southwell et al., 2019; Wang et al., 2019). Such concerns are currently high on the agenda, given the challenge of vaccine hesitancy in the struggle against the Covid-19 pandemic (Johnson et al., 2020). At the same time, digital platforms have proven to have positive health effects, by providing social and emotional support, enabling the exchange of advice and information (Kendal et al., 2017; Lupton, 2017; Maslen and Lupton, 2019), and increasing self-care (Grosberg et al., 2016; Smith et al., 2015). The Internet’s potential to reduce socioeconomic inequalities in health, through information-sharing and peer support online, has also been pointed out (Grosberg et al., 2016). Relatedly, the movement towards ‘epistemic democracy’ (Lynch, 2017), through which expert knowledge increasingly competes or co-exists with other knowledge claims due to the democratization of the ability to access and spread information, increases the possibilities for marginalized groups to voice concerns and partake in knowledge production (Rentschler, 2014). While epistemic democratization has been pointed out as paving the way for a ‘post-truth’ era (Budgeon, 2021; Lynch, 2017), the ‘misinformation’ model, assuming a sharp epistemic divide between established medical expertise and lay knowledge production online, has also been placed in question (Maslen and Lupton, 2019; Vuolanto et al., 2020). Scholarly attention has been directed at the ‘expert patient’ and a ‘blurring of the lines between lay and expert knowledges and scientific and non-scientific expertise in the health and medical realm’ (Maslen and Lupton, 2019: 1637).

This article contributes to research on lay knowledge-building about health in the Internet era, through the case of a group of women claiming that their use of the contraceptive copper intrauterine device (IUD) has led to systemic side effects, some of them severe. While established medical expertise holds that the copper IUD may lead to increased menstrual pain and bleeding, this group of women reports a range of side effects encompassing both mental and physical symptoms, such as depression, irritability, fatigue, skin problems and loss of hair, claimed to result from copper-related systemic disturbances and chronic inflammation caused by the IUD. While such references to systemic side effects of the copper IUD have an international reach, and have been noted in passing in previous research (Amico et al., 2020; Dehlendorf et al., 2014; Nguyen and Allen, 2018; Wemrell and Gunnarsson, 2022b), the focus of this article is the Swedish context, where the alternative knowledge claims about the copper IUD pivot around a Facebook group formed in 2014 and presently gathering 8600 members (in a country with around 10 million inhabitants).

Based on online group interviews and written essays collected from members of the Facebook group, this article examines how these women navigate and negotiate various sources of information when building their knowledge claims, with a focus on the role of scientificity in these epistemic negotiations. In line with previous health movements and activists, embodied, ‘experiential knowledge’ (Epstein, 2011: 266) comprised a fundamental building block of our participants’ knowledge claims. However, to be able to draw systematic conclusions from their collectively processed experiences, they drew on a range of pre-existing discursive resources available online, ranging from blog posts to scientific articles, and it is this aspect of the women’s epistemic proceedings that is the concern of this article. Building on research questioning the dualistic juxtaposition of ‘scientific’ established knowledge and ‘non-scientific’ lay knowledge (Epstein, 2011; Goldenberg, 2016; Shaw, 2002; Vuolanto et al., 2020; Wynne, 2006), we show that whereas the representatives of medical authorities tend to portray the claims of these women as unscientific, many participants were strongly invested in scientificity and criticized the knowledge claims of medical authorities for *not being scientific enough*. Meanwhile, due precisely to a perceived lack of scientifically based expertise on their condition, including evidence-based diagnostic procedures and treatments, many of the women reported having little choice but turn to less scientific modes of health-related knowledge and practices.

In the following, we provide an overview of research on lay knowledge-building on health, drawing on work from the overlapping fields of health movements and activism and the lay contestation of health-related expertise in an online context. We then present the background and research design of our study. The analysis is divided into a section focusing the role of scientificity in the women’s epistemic proceedings, and a section examining why alternative healthcare practices become pivotal even for participants strongly invested in scientificity.

## 1. Previous research on lay knowledge-building on health

We situate the epistemic practices of the group analysed in this article within the research field studying health movements and activism, where the contestation of medical expertise and building of counter-knowledges are key themes (Brown et al., 2004; Epstein, 2011; Landzelius, 2006; Tomes and Hoffman, 2011; Zavestoski et al., 2004), and within the related field addressing lay contestations of expertise in an online context (Goldenberg, 2016; Nisbet and Scheufele, 2009; Southwell et al., 2019). A central observation made by researchers in these overlapping fields is that during the last half century the relationship between patients and medical authorities has gone through significant shifts. Tomes and Hoffman (2011) succinctly summarize this development:

What the physician-ethicist Jay Katz described in 1984 as the ‘silent world of doctor and patient’, characterized by the latter’s ‘surrender to silent and blind trust’ in the former, seems far distant from today’s reality. Inspired by civil rights, feminist, and consumer activism, a variety of patient empowerment movements have fomented a noisy revolt against medical authority. (pp. 1–2)

Thus, while the trend of lay people increasingly contesting medical authorities has been accentuated in the Internet era, it has older roots in the general social movement culture emerging in the 1960s (Brown et al., 2004). Health movements have been fuelled by various patient groups’ collectively articulated personal experiences of illness and their complaints about the knowledge base and treatment strategies of established medical institutions (Brown et al., 2004; Epstein, 2011; Landzelius, 2006; Tomes and Hoffman, 2011; Zavestoski et al., 2004).

Brown et al. (2004) list three reasons for the increased prevalence and significance of health movements challenging medical authorities. First, they emphasize the success of the women’s health movement in changing medical practices and understandings of women’s health issues. As part of the broader 1970s feminist movement, the pressure from women’s health activists ‘greatly altered medical conceptions of women, broadened reproductive rights, expanded funding and services in many areas, altered many treatment forms (e.g. breast cancer), and changed medical research practices’ (Brown et al., 2004: 51), thereby paving the way for health movements in other areas. Second, the authors underscore the emergence of a broader health awareness culture, which other scholars have linked to new forms of health consumerism (Leahy et al., 2011; Tomes and Hoffman, 2011). Smith and Graham (2019) highlight the contradictory facets of the ‘consumer autonomy’ with regard to health, which is increasingly encouraged in the neoliberal area: ‘patients are expected and have been encouraged to take an active role in managing their own health’, but ‘the growth of patient “consumer autonomy” has also created “flattening” of expertise, where power shifts from doctors to patients’ (p. 1312). Third, already in 2004 Brown and colleagues identified the Internet as a central facilitator of health movements. With the emergence and development of user-driven social media platforms, enabling people to share information and connect in ever faster and simpler ways, the significance of Internet technology has been clearly accentuated (Maslen and Lupton, 2019; Smith and Graham, 2019). Women in particular have been found to rely on the Internet for information about health (Bidmon and Terlutter, 2015; Fox and Rainie, 2002).

As highlighted earlier, feminist health activism has been central in the trend of challenging medical authority, and contraceptive technologies and their user-friendliness have here been key issues, alongside childbirth policy and abortion (Tomes and Hoffman, 2011). As an example, in the 1970s, the Dalkon Shield Information Network formed around the severe and sometimes lethal side effects attributed to the Dalkon Shield IUD. It contributed towards product recall in the United States and paved the way for litigation over the Copper-7 IUD in 1988 (Dugdale, 1995; Grant, 1992). Apart from issues directly related to women’s reproductive health, health movements centring on ‘contested disorders’ (Landzelius, 2006) or ‘contested illnesses’ (Dumit, 2006), such as fibromyalgia and chronic fatigue, have often also been women’s health movements, since such disorders affect women more often than men (Yunus, 2001).

Health movements and patient groups organizing to resist and negotiate medical authority have an ambiguous relationship to science. In research and public discussion, resistance to or contestation of medical scientific expertise, not least online, is often framed in terms of misinformation or lay knowledge deficits (Southwell et al., 2019; Wang et al., 2019). A growing body of literature shows, however, that this ‘misinformation’ model does not adequately reflect the nature of ‘lay knowledge’ (Shaw, 2002) or map what is actually or always going on when lay people challenge medical authorities (Goldenberg, 2016; Nisbet and Scheufele, 2009; Vuolanto et al., 2020; Wynne, 2006). It is indeed the case that health movements draw largely on ‘embodied experience as counter-authority’ (Zavestoski et al., 2004: 269), in alignment with the noted influence from feminist movements (Tomes and Hoffman, 2011). However, far from being simply against or ignorant of science as such, health movements have often placed the demand for scientific and judicial information, and objections to the inadequacy or withholding of such knowledge or information, at the centre of their struggles (e.g. Dugdale, 1995; Grant, 1992).

Epstein (2011) argues that rather than resisting biomedical science, patient groups and health movements ‘have combined experiential knowledge with varying degrees of mastery of formal knowledge, often producing interestingly hybrid or “translocal” ways of knowing or varieties of expertise’ (p. 266; cf. Kangas, 2001). Similarly, in their study of a Facebook women’s health group, Maslen and Lupton (2019) show that those with an ‘expert position’ within the group were highly invested in ‘biomedical ways of knowing’ (p. 1647) even while contesting biomedical authorities and complementing scientific knowledge claims with experiential knowledge. In line with this, Vuolanto et al. (2020), who studied groups engaged in what is commonly referred to as ‘alternative’ health practices, show that rather than being the other of biomedical science, this sort of practice ‘*both* aligns itself with biomedical expertise *and* seeks to transform it’ (p. 512) in a way that destabilizes the ‘alternative-biomedical boundary’ (p. 509). Thus, while non-scientific content regarding health-related matters, aligning with a ‘post-truth’ condition where ‘objective facts are less influential in shaping public opinion than appeals to emotion and personal belief’ (Oxford Dictionaries cited in Lynch, 2017: 594), can obviously be located online, research has established that an anti-scientific approach is not the only driving force behind health movements’ contestation of medical expertise. Such groups have criticized medical scientific expertise on grounds that are often themselves scientifically oriented.

## 2. This study

The participants in this study are women suspecting or convinced that the use of copper IUDs can cause illness, of whom almost all had personal experiences of unwellness that they associated with the copper IUD. They were recruited from a Facebook group, founded in 2014, which organizes the Swedish branch of a larger international movement centred on the issue of copper excess due to copper IUD use.^1^ We situate the group at the informal and loosely organized end of Landzelius’ (2006) broad notion of health activism, including a spectrum from formalized patient groups allying with policy bodies to effectuate change (e.g. Rabeharisoa et al., 2014) to ‘quasi-organized loose networks linked by-and-large via gestures of solidarity and co-identification’ (Landzelius, 2006: 532). While the group’s activities are ultimately driven by its members’ quest for a cure, this personal issue is interwoven with the collective or social issue (Zavestoski et al., 2004) formed by the gathering of members, and inextricably implicated in an epistemo-political process of challenging medical authorities and its power. As noted by Landzelius (2006), ‘people suffering from disorders that are contested or denied *must* organize, to literally author into existence “their” respective diseases’ (p. 532; cf. Dumit, 2006).

The Facebook group description declares that it is for persons who suspect that they suffer from ‘copper excess/copper intoxication’, with or without a copper IUD, but side effects of the copper IUD is the key focus. Symptoms listed in the description are numerous, including mental/emotional issues such as anxiety, irritability, panic attacks, depression and sleeping problems, and physical symptoms such as heart palpitations, joint pain, nausea, drastic loss of hair, weight gain, stomach pain, hypersensitivity to sounds, skin problems and exhaustion. The symptoms are claimed to result from systemic effects of copper, leading, for example, to neurological and hormonal imbalances, and chronic inflammation caused by the IUD.

The Facebook group description states that it offers a space for ‘discussing symptoms, detoxication and how to get well again’. It is used for sharing information, advice and personal experiences, alongside providing emotional support. The group has also been involved in mobilization around the issue, through contacts with the media and through a concerted effort to engage the Swedish Medical Products Agency by encouraging group members to report suspected side effects of the copper IUD to the agency. The high number of complaints led the agency to carry out an investigation, which concluded, in 2018, that there is no scientific evidence supporting the women’s claims and that it is highly unlikely that the levels of copper released from the IUD could cause the symptoms described (Swedish Medical Products Agency, 2018).

The question driving this study was how women adhering to these knowledge claims about the copper IUD navigate competing claims about the issue and build their own view. To explore this, we conducted seven online group interviews with a total of 23 women recruited from the Facebook group (2–4 persons/group) and collected 23 written essays from members of the same group. With permission from the group administrators, Gunnarsson posted an inquiry describing the aim of the study and inviting participants who ‘are convinced or suspect that the copper IUD may lead to a range of side effects commonly unacknowledged by conventional healthcare’ to groups interviews. Upon the request from some women interested in participating but not in the interview format, written essays about how members came to be convinced of or suspect the unestablished side effects were also invited. Twenty-three essays were collected, of which 11 were written by women also participating in an interview. While no sociodemographic data were gathered for the essay participants, the women we interviewed were 20–56 years old. Most had attained tertiary education. They worked as teachers, entrepreneurs, managers, doctoral researchers, blue collar workers, medical doctors and nurses, in the complementary health sector, with media and communication and in the beauty sector.

The interviews were carried out via Zoom during Fall 2020, with Gunnarsson as convener and Wemrell as co-convener. The interviews followed a semi-structured format, centred on questions about how participants came to adhere to their epistemic standpoints regarding the copper IUD, how they navigated the informational landscape in this process, and their experiences of relating to healthcare regarding the issue. A considerable part of the interviews was dedicated to each participant’s recounting of their personal story. Despite the online format, a significant degree of group interaction also characterized the interviews, arguably encouraged by the commonality and societal marginality of most participants’ experiences. The interviews were conducted in Swedish and lasted for 1.5–2.5 hours. The study was approved by the Swedish Ethical Review Authority (no. 2019-03017). Interview transcripts and essays were processed in NVivo, using a combination of data-driven and concept-driven coding, and analysed thematically (Braun and Clarke, 2006).

All essay participants and 21 out of 23 group interview participants had personal experiences of health problems that they associated with using a copper IUD. The remaining two participants were committed to the issue due to their professional activities in the complementary health sector. Among participants with personal experiences some reported having made visits to the emergency ward and fearing they suffered from a mortal disease, while others spoke of diffuse and relatively mild, although long-lasting, mental and physical symptoms.

While a few participants suspected the copper IUD to be the cause of their health problems before coming across the alternative knowledge claims, most did not make this connection until finding the Facebook group or other sources communicating the alternative discourse. Since the noted symptoms overlap with those of other conditions, finding these sources was reported to be challenging. It was described as requiring Internet searches for several symptoms at the same time, or including ‘copper IUD’ in the search after having heard about the alternative discourse from someone else. Finding the Facebook group was commonly referred to as an experience of things falling into place: ‘the penny just dropped’ was an expression used by several participants. Participants could identify with the experiences and symptoms shared by others, and started making connections between their copper IUD use and their health problems. Some participants reported a distinct weakening or disappearance of symptoms shortly after removing their copper IUD, while others spoke about a slower recovery or problems remaining. While a few referred to positive experiences of interaction with healthcare professionals, most participants spoke about not having been taken seriously, sometimes to the point of being ridiculed or laughed at, when raising their suspicion towards the copper IUD as the cause of their health problems. Several had been prescribed anti-depressant medication or referred to a psychologist.

## 3. The collective labour of scientific patchworking

A clear feature of the data was that, rather than uncritically adopting any kind of information on the copper IUD, many participants described having engaged in a time-consuming labour of identifying, assessing and drawing conclusions from various sources including scientific ones. The scientific orientation and critical skills arguably varied in the group of women adhering to the alternative discourse on the copper IUD, but our participants generally displayed a strong investment in scientificity, both in the sense that they tended to prioritize information based on scientific research and that their judgements were broadly guided by scientific principles such as logicality and systematicity (Xu, 2005). Some participants particularly, occupying an ‘expert’ position within the community (cf. Maslen and Lupton, 2019), voiced a complex set of scientifically oriented arguments drawn on to build their claim about systemic side effects. This is in line with previous research pointing to critical engagement with health information on the Internet (Henderson and Eccleston, 2013).

The most central scientifically oriented argument, used in the face of medical authorities stating that no scientific studies support the existence of such side effects, was simply that neither do any scientific studies *reject* such side effects. Responding to the Swedish Medical Products Agency report on the copper IUD, Amanda, a medically trained interview participant (G1, 34 y.), stated,

[T]here’s no scientific evidence supporting that it’s harmless . . . the studies are lacking and what they say is that no connection can be confirmed. But what they write makes it sound as if there were no connection. And that’s two totally different things.

Sissela (G2, 40 y.) underlines that the burden of proof should lie on medical authorities, not the women (cf. Zavestoski et al., 2004):

They are the ones who need to present evidence. We should not have to prove that this is because of the copper IUD, they must prove that this is harmless.

In the group interviews, participants were asked to respond to an excerpt from a document providing guidelines for health personnel administering contraceptive devices, where none of the systemic side effects reported by the women are mentioned. Maria (G1, 37 y.) noted that in the guidelines, it appears as if there is scientific evidence proving that systemic side effects do not exist:

They asked only about bleeding, how many got pregnant, how often it [the IUD] fell out and if there was more pain. And they got these results because that’s the only thing they asked about. . . They should have written that no studies have been made on systemic side effects or the like, shouldn’t they?

While ‘expert’ participants referred to some existing research on links between IUD use and blood copper levels (de la Cruz et al., 2005), some related the paucity and perceived deficiency of research to medical devices, including copper IUDs, not being subject to the same rigorous controls as pharmaceutical drugs (see Maak and Wylie, 2016). To the women, this basically meant that the data supporting the safety of copper IUDs do not live up to scientific standards.

Alongside pointing to an absence of scientific study of the claimed side effects, the participants drew on scientifically based information from related medical areas. Such topics included symptoms of copper intoxication due to, for example, excessive oral consumption or being cut by a copper item, and of Wilson’s disease, a rare genetic disorder causing an accumulation of copper in the body, both of which were said to align with symptoms reported by women. In addition, scientific support was claimed from nutritional science addressing the interplay between minerals and hormones in the body, indicating that levels of copper can affect the hormonal balance in ways that may explain many of the symptoms reported by the women.

Matilda (G3, 34 y.) summarized what we see as the principal scientifically oriented idea behind the claim about systemic side effects:

There’s loads of research on why these side effects are plausible. But there are no specific studies on their connection to the copper IUD.

The women also challenged the scientific validity of established assumptions about mechanisms pertaining to the copper IUD, most notably that the release of copper inside the body is non-toxic as it is smaller than the daily intake of the mineral through food and water. This statement, which has been put forward by the Swedish Medical Products Agency (2018), was challenged by participants arguing that oral uptake and release of copper into the blood or body tissue are two different things.

The Facebook group and other Internet platforms served as forums where information was shared and digested second hand, but several participants also spoke about going directly to scientific sources. Amanda, who had been severely ill due, in her opinion, to her copper IUD, was initially sceptical towards the alternative claims about the copper IUD. However, upon strongly identifying with the stories shared in the Facebook group, and reading other members’ posts about the interaction between copper and the hormonal system, she started looking for research on the topic:

There were very knowledgeable persons who could tell me about how the hormones are affected. . . and I haven’t learnt about this at all during my [medical] education, so it was totally new to me. But then, when I started looking for studies, I found studies on how, for example, copper and oestrogen affect one another.

Many reported about an intense labour of searching for *any* sort of clue to why they felt as they did, a scanning work that often included, while not being limited to, scientific resources:

I have God damn gone over everything, I mean how hormones affect one another, how different substances affect. . . I have vacuum cleaned the web, I mean I’m the kind of person who reads research reports and shit, all sorts of things (Ann-Kristin, G2, 36 y.)

I have read so many research reports, I have read so many articles. . . I’ve gone over how the liver works, how detoxication works, how the intestinal flora works, how the brain works. I have practically read around the clock during four years’ time to find out how to get well. (Elisabeth, G4, 52 y.)

Melinda (G2, 35 y.) underscored the research-like, critical labour required to navigate the informational landscape:

You have to check where information comes from, it’s not like I just read something and then just ok, fine, this is it. . . That requires a lot of time and commitment. It takes time to read research reports, it takes time to analyse it. . . You’re sort of like a little researcher yourself, but without a title [laughs].

Given what they saw as a scientific commitment among the Facebook group members, several participants took issue with the categorization of their claims as non-scientific.

As expressed by an essay participant,

I get the feeling that healthcare sees this Facebook group as one where ‘alternative facts’ are spread. As a member I rather get the feeling that many of us have read and discussed the scientific studies that exist to a much larger extent than the average gynaecologist, midwife or medical doctor. (E11)

Along similar lines, some participants pointed to a perceived lack of critical thinking, or of ‘taking in information that goes a bit outside of what they have learnt’ (Ann, G7, 38 y.) among some healthcare staff. Amanda, the medically trained interview participant, said her illness journey had changed her view of the relationship between scientific truth and what one learns as a medical doctor or student:

This has really made me develop professionally. Just because it doesn’t say so in a book it doesn’t mean it’s not like that. I have become even more critical of different drugs and treatments that we give just because.

Although it is not our task to judge the truth value of the women’s claims about side effects of the copper IUD, the analysis in this section shows that depictions of these claims as anti-scientific ‘alternative facts’ (Marion, 2018) are inadequate. We propose the notion of a *collective labour of scientific patchworking* to denote that process, heavily underpinned by digital information-sharing, through which the women identify, assess and inter-relate existing scientific resources deemed relevant to their cause, broadly based on scientific principles such as systematicity and logicality. Rejecting the label as ‘the other of science’ (Vuolanto et al., 2020: 513), the women turned the tables, arguing that medical authorities and healthcare were the ones that were not scientific enough.

## 4. Pinched in-between the established and the alternative

While scientificity centrally guided our participants’ epistemic proceedings, they also turned to less scientific informational sources and practices, aligning with what Vuolanto et al. (2020) refer to as a practice of ‘“mi[xing]-and-match[ing”] biomedical and alternative modes of knowledge’ (p. 512). While establishing that using a copper IUD may lead to systemic side effects was an important part of the women’s commitment, the ultimate driving force behind their epistemic struggle was the wish to get well. This goal made them invested in accessing and evaluating information not only on how to explain their illness but also how to recover from it, information they reported being unable to obtain from conventional healthcare providers, who typically did not acknowledge any systemic side effects of the copper IUD. The issue of diagnosis here became pertinent, and a matter of concern stressed by the women was the absence of scientifically established means for measuring copper excess in the bodily system as a whole.

There are, however, alternative or complementary medicine (CAM) practitioners claiming to be able to measure copper levels in the body through so-called hair mineral analysis. Some participants had tried this, and in cases where a copper excess was identified, mineral supplements aimed at rebalancing and detoxicating the body were commonly prescribed. This procedure lacks a solid scientific evidence-base, though (Seidel et al., 2001). Hence, regarding the issue of diagnosis and recovery, the women could not reach out for scientifically valid information and practices in the same way as when bolstering their claim about the plausibility of systemic side effects from the copper IUD. How was this tension negotiated by participants invested in scientificity?

Although some participants had previous experiences of CAM, many had not, and some declared having been decisively against alternative frameworks before their copper IUD experience. These women, who expressed a high confidence in science and, before their copper IUD experience, in medical authorities, are particularly interesting here, since their experiences accentuate the complex relationship between the alternative and the scientific.

Rebecka (G4, 36 y.), stating ‘I want all the decisions I take to have some kind of scientific basis’ and that she ‘really had issues with alternative methods before’, spoke about trying out CAM methods by lack of conventional ones. This theme was echoed by others. The participants’ use of CAM practices ranged from consulting professional practitioners to experimenting themselves with cures and supplements they had read about. While many were pleased with the results, several expressed disappointment and distrust. Alongside the scientific deficit, the high cost of CAM counselling was repeatedly raised as undermining trust. Amanda articulates this issue, while highlighting the gap filled by the CAM practitioners:

I’m open to the fact that there are other alternatives, absolutely, but when it ends up costing so much money, you’re prescribed loads of supplements – on what is that based?. . . At the same time, I see that there is that need. People need to have an answer, people need to understand.

The theme of women being pinched in-between conventional and alternative practitioners, both seen as lacking in credibility, was central in our data:

You end somewhere in-between, reaching out to healthcare with the risk of not being believed and of being questioned, and also they don’t seem to have any good measurement methods, and on the other side connecting with someone who makes a hair mineral analysis, and then you get a package with a whole lot of pills from an American firm, and you don’t know anything about what they do with your body. And there’s loads of money to be earned on these hair mineral analyses and pills. . . How are you supposed to navigate that?. . .

Interviewer: You can’t trust regular healthcare, but you can’t really trust these alternative actors either?

Yes, exactly. (Marianne, G3, 45 y.)

Some pointed out how conventional healthcare’s non-acknowledgement of the women’s perspectives pushed women into the CAM sector and into experimenting on their own:

This is where we end up when healthcare does not take women’s testimonies seriously and does not want to investigate it in a serious way. Then, in this alternative sphere of therapeutic methods there’s the whole spectrum of practitioners all of which are not serious and definitely cannot guarantee that it is well-grounded, reliable. . . Experimenting with these things feels kind of precarious. But that’s what we’re left with when we don’t get serious advice. (Linda, G7, 36 y.)

When seeking to establish the plausibility of systemic copper IUD symptoms, the women were able to reach out for scientific support, however fragmented and incomplete. However, in this section, we have shown that in their quest for ways to recover, they had difficulties identifying scientific foundations for what to try out and believe in. Rather than *choosing* to try CAM practices with limited scientific foothold, many participants felt *compelled* to do so by lack of options. Although both conventional and CAM practitioners were seen as *not scientific enough*, the latter were deemed more attractive since, unlike conventional healthcare, they did acknowledge the women’s own beliefs about the reasons for their health problems.

## 5. Conclusion

Building on the growing number of studies questioning the dualistic juxtaposition of ‘scientific’ medical authorities and ‘non-scientific’ lay knowledge (Epstein, 2011; Goldenberg, 2016; Shaw, 2002; Vuolanto et al., 2020; Wynne, 2006; Zavestoski et al., 2004), in this article, we have addressed the role of scientificity in the epistemic proceedings of members of a Swedish women’s health group pivoting around the issue of suspected systemic side effects of copper IUD use. While collectively processed embodied experiences were a fundamental basis of their epistemic proceedings, and alternative modes of knowledge with a weak scientific basis were drawn on, the women’s critique of medical authorities and conventional healthcare centrally hinged on the claim that these were *not scientific enough*. We here propose the notion of *collective labour of scientific patchworking* to capture our participants’ endeavours of identifying, assessing and inter-relating existing scientific resources deemed relevant to their cause. Apart from underscoring the *scientific* resources and scientific-minded approach broadly guiding this scientific patchworking, the notion emphasizes the time-consuming and demanding *labour* that people suffering from contested forms of illnesses (Dumit, 2006; Landzelius, 2006) are pushed into performing when established expertise fails to meet their needs. Also, although our participants reported about strongly individualized modes of navigating the online informational landscape, the notion draws attention to the *collective* embedding of these individual strategies. The scientific patchworking that the women described was a complex endeavour in that it drew on a range of scientifically complex sources that had to be assessed and interrelated, a project that depended on a collaborative sharing and assessment of information. It is likely that the participants in the study, who were overall highly educated, are more engaged and scientifically oriented than the average adherent of the knowledge claims disseminated in the Facebook group. However, the more engaged and scientifically oriented women, some of whom participated in our study, to various degrees acquired ‘expert positions’ within the community (cf. Maslen and Lupton, 2019), thereby heavily shaping the epistemic framework of the group as a whole. The collective labour of scientific patchworking observed in this study can be seen as a lay epistemic means of compensating for a zone of ‘undone science’ (Hess, 2009), which, had it been interpreted as such by policy bodies and researchers, may have led to ‘the opening of new complex scientific questions’ potentially advancing scientific medical knowledge (Rabeharisoa et al., 2014: 119).

Our participants’ scientific patchworking was one key component in a more encompassing labour of epistemic patchworking also involving collectively processed personal experiences as well as sources with a weak scientific basis. Our study aligns with previous research on lay knowledge-building on health in that the women moulded their frameworks from ‘bits and pieces taken from many sources’, reflecting ‘an increasingly plural and complex environment of knowledge’ (Kangas, 2001: 89; cf. Epstein, 2011; Vuolanto et al., 2020). However, while this form of mixing and matching is often associated with a ‘diminishing faith in science’ (Kangas, 2001: 89), a central contribution of this study is that even the most scientifically oriented participants were drawn to less scientific information and practices, not because of a lack of trust in science but due precisely to what they saw as conventional healthcare’s lack of scientifically updated knowledge on their condition and, hence, inability to help them. Thus, engagements with the alternative resources did not necessarily derive from a non- or anti-scientific disposition.

Many studies on attitudes towards IUDs emphasize the importance of countering misinformation and rumours expressed via social networks, including social media (Daniele et al., 2017). Such perspectives, often mirroring standpoints taken by medical authorities, risk simplifying the nature of lay knowledge-building, thereby deepening divides between patients/lay people and medical authorities. Goldenberg (2016) takes issue with the tendency of biomedical and policy authorities to frame public resistance to biomedical expertise as simply ‘a conflict of *science versus ignorance*, the former unproblematic and the latter entirely flawed’ (p. 554; cf. Wynne, 2006), underlining that that this dualistic framing is liable to undermine the goal of authorities to conciliate public health agendas with the concerns of lay people through increasing, rather than decreasing, polarization and public mistrust (Nisbet and Scheufele, 2009).

While contemporary societies face great challenges in managing conspiracy theories and the dissemination of ‘alternative facts’, it is clearly problematic to categorize all forms of knowledge claims challenging epistemic authorities as simply irrational and non- or anti-scientific. ‘Science’ and ‘non-science’ are no mutually isolated entities, given that scientific knowledge continually evolves, in dialogue with broader social developments and activities. There are many examples of scientific controversies (de Freitas and Pietrobon, 2010; Epstein, 1996) and of health-related knowledge claims that were previously rejected but are today scientifically established, not least in the women’s health sector and with regard to risks associated with contraceptives (e.g. Dugdale, 1995; Grant, 1992). Loosening the dualistic scientific/non-scientific scheme, acknowledging the complexity and imperfection of scientific underpinnings of medical practice, is important for narrowing the gap between conventional and alternative knowledge claims and recovering trust in medical authority.

There are indeed examples of patient groups’ perspectives having been embraced by research and policy institutions in ways that not only fostered patients’ trust and feelings of being respected but also advanced medical knowledge (Epstein, 1996; Rabeharisoa et al., 2014). While Rabeharisoa et al. (2014) distinguish health activists working ‘from within’ dominant institutions from health movements, which ‘contest institutions from the outside’ (p. 111), we contend that this is another binary that should be destabilized. Within this inside/outside scheme the group studied in this article would count as oppositional to rather than collaborative with medical authorities, but this, we argue, seems to have less to do with the approach of the group itself than with the framing of their claims as ‘misinformation’ rather than as potentially valuable input that might spur scientific advances.

In the interest of patient care and of decreasing public mistrust in medical and scientific institutions, a constructive response to knowledge claims such as those addressed in this study should combine the evaluation of the claims with ‘honest institutional-scientific self-reflective questioning’, including a critical stance towards representations of current medical guidelines as static and infallible ‘factual scientific truths’ (Wynne, 2006: 216, 217). As Wynne (2006) notes, despite widely dispersed commitments to two-way models of ‘public engagement in science’, there is a persistent tendency for institutions to manage public distrust by simply reinforcing the message that the public is mistaken. However, solving the problem of mistrust also involves maintenance of the trustworthiness of institutional science and healthcare, through reflection on how these may be imbricated in the issue (Wemrell and Gunnarsson, 2022a, 2022b; Wynne, 2006), including the recognition of uncertainty (Shackley and Wynne, 1996) and of past (perceived) mistakes (Chrismas, 2012).

The current ‘post-truth’ condition, where many counter-establishment knowledge claims are based on affective dispositions disconnected from any serious quest for knowledge (Budgeon, 2021; Lynch, 2017), raises new challenges for the prospect of bridging lay and established knowledge. It risks diminishing the possibilities for groups such as the one studied in this article to be taken seriously and is likely to reinforce polarizing representations of ‘anti-scientific’ lay knowledge versus ‘scientific’ established knowledge. In this specific historical setting there is hence a strengthened need for epistemic authorities to examine counter-knowledges with care, self-reflexivity and openness, to be able to distinguish reasonable contestations of established knowledge claims from unreasonable ones.

## Supplemental Material

sj-docx-1-pus-10.1177_09636625221107505 – Supplemental material for On the verge between the scientific and the alternative: Swedish women’s claims about systemic side effects of the copper intrauterine device Click here for additional data file. Supplemental material, sj-docx-1-pus-10.1177_09636625221107505 for On the verge between the scientific and the alternative: Swedish women’s claims about systemic side effects of the copper intrauterine device by Lena Gunnarsson and Maria Wemrell in Public Understanding of Science

## Notes

1 The Swedish Facebook group is comparatively large but similar groups exist in other languages. The website https://coppertoxic.com is a central English-language resource on the topic.

## References

[bibr1-09636625221107505] Amico JR Stimmel S Hudson S Gold M (2020) ‘$231 . . . to pull a string!!!’ American IUD users’ reasons for IUD self-removal: An analysis of internet forums. Contraception 101(6): 393–398. 32088175 10.1016/j.contraception.2020.02.005

[bibr2-09636625221107505] Bidmon S Terlutter R (2015) Gender differences in searching for health information on the internet and the virtual patient-physician relationship in Germany: exploratory results on how men and women differ and why. Journal of Medical Internet Research 17(6): e4127. 10.2196/jmir.4127 PMC4526954 26099325

[bibr3-09636625221107505] Braun V Clarke V (2006) Using thematic analysis in psychology. Qualitative Research in Psychology 3(2): 77–101.

[bibr4-09636625221107505] Brown P Zavestoski S McCormick S Mayer B Morello Frosch R Gasior Altman R (2004) Embodied health movements: New approaches to social movements in health. Sociology of Health & Illness 26(1): 50–80. 15027990 10.1111/j.1467-9566.2004.00378.x

[bibr5-09636625221107505] Budgeon S (2021) Making feminist claims in the post-truth era: The authority of personal experience. Feminist Theory 22(2): 248–267.

[bibr6-09636625221107505] Chrismas R (2012) The people are the police: Building trust with Aboriginal communities in contemporary Canadian society. Canadian Public Administration 55(3): 451–470.

[bibr7-09636625221107505] Daniele MA Cleland J Benova L Ali M (2017) Provider and lay perspectives on intra-uterine contraception: A global review. Reproductive Health 14(1): 1–11. 28950913 10.1186/s12978-017-0380-8 PMC5615438

[bibr8-09636625221107505] de Freitas RS Pietrobon R (2010) Why care about scientific controversies? Continuities and discontinuities in the history of science. Journal of Historical Sociology 23(4): 501–516.

[bibr9-09636625221107505] de la Cruz D Cruz A Arteaga M Castillo L Tovalin H (2005) Blood copper levels in Mexican users of the T380A IUD. Contraception 72(2): 122–125. 16022851 10.1016/j.contraception.2005.02.009

[bibr10-09636625221107505] Dehlendorf C Kimport K Levy K Steinauer J (2014) A qualitative analysis of approaches to contraceptive counseling. Perspectives on Sexual and Reproductive Health 46(4): 233–240. 25040686 10.1363/46e2114 PMC4487742

[bibr11-09636625221107505] Dugdale A (1995) Devices and Desires: Constructing the Intrauterine Device, 1908-1988. PhD Thesis, University of Wollongong, NSW, Australia.

[bibr12-09636625221107505] Dumit J (2006) Illnesses you have to fight to get: Facts as forces in uncertain, emergent illnesses. Social Science & Medicine 62(3): 577–590. 16085344 10.1016/j.socscimed.2005.06.018

[bibr13-09636625221107505] Epstein S (1996) Impure Science: AIDS, Activism, and the Politics of Knowledge. Oakland, CA: University of California Press. 11619509

[bibr14-09636625221107505] Epstein S (2011) Measuring success scientific, institutional, and cultural effects of patient advocacy. In: Hoffman B Tomes N Grob R Schlesinger M (eds) Patients as Policy Actors. New Brunswick, NJ: Rutgers University Press, pp. 257–277.

[bibr15-09636625221107505] Fox S Rainie L (2002) Vital decisions: How Internet users decide what information to trust when they or their loved ones are sick. Pew Internet and American Life Project. Available at: http://www.pewinternet.org/reports/pdfs/PIP_Vital_Decisions_May2002.pdf

[bibr16-09636625221107505] Goldenberg MJ (2016) Public misunderstanding of science? Reframing the problem of vaccine hesitancy. Perspectives on Science 24(5): 552–581.

[bibr17-09636625221107505] Grant N (1992) The Selling of Contraception: The Dalkon Shield Case, Sexuality, and Women’s Autonomy. Columbus, OH: Ohio State University Press.

[bibr18-09636625221107505] Grosberg D Grinvald H Reuveni H Magnezi R (2016) Frequent surfing on social health networks is associated with increased knowledge and patient health activation. Journal of Medical Internet Research 18(8): e212. 10.2196/jmir.5832 PMC4997002 27511272

[bibr19-09636625221107505] Hardey M (1999) Doctor in the house: The Internet as a source of lay health knowledge and the challenge to expertise. Sociology of Health & Illness 21(6): 820–835.

[bibr20-09636625221107505] Henderson EM Eccleston C (2013) An online adolescent message board discussion about the internet: Use for pain. Journal of Child Health Care 19: 412–418. 24270989 10.1177/1367493513509420

[bibr21-09636625221107505] Hess DJ (2009) The potentials and limitations of civil society research: Getting undone science done. Sociological Inquiry 79(3): 306–327.

[bibr22-09636625221107505] Johnson NF Velásquez N Restrepo NJ Leahy R Gabriel N El Oud S , et al. (2020) The online competition between pro- and anti-vaccination views. Nature 582(7811): 230–233. 32499650 10.1038/s41586-020-2281-1

[bibr23-09636625221107505] Kangas I (2001) Making sense of depression: Perceptions of melancholia in lay narratives. Health 5(1): 76–92.

[bibr24-09636625221107505] Kendal S Kirk S Elvey R Catchpole R Pryjmachuk S (2017) How a moderated online discussion forum facilitates support for young people with eating disorders. Health Expect 20: 98–111. 26725547 10.1111/hex.12439 PMC5217921

[bibr25-09636625221107505] Landzelius K (2006) Introduction: Patient organization movements and new metamorphoses in patienthood. Social Science & Medicine 62(3): 529–537. 16054282 10.1016/j.socscimed.2005.06.023

[bibr26-09636625221107505] Leahy M Löfgren H de Leeuw E (2011) Introduction: Consumer groups and the democratization of health policy. In: de Leeuw E Leahy M (eds) Democratizing Health. Cheltenham: Edward Elgar, pp.1–14.

[bibr27-09636625221107505] Lupton D (2017) Digital Health: Critical and Cross-Disciplinary Perspectives. London: Routledge.

[bibr28-09636625221107505] Lynch M (2017) STS, symmetry and post-truth. Social Studies of Science 47(4): 593–599. 28791930 10.1177/0306312717720308

[bibr29-09636625221107505] Maak TG Wylie JD (2016) Medical device regulation: A comparison of the United States and the European Union. Journal of the American Academy of Orthopaedic Surgeons 24(8): 537–543. 27195383 10.5435/JAAOS-D-15-00403

[bibr30-09636625221107505] Madathil KC Rivera-Rodriguez AJ Greenstein JS Gramopadhye AK (2015) Healthcare information on YouTube: A systematic review. Health Informatics Journal 21(3): 173–194. 24670899 10.1177/1460458213512220

[bibr31-09636625221107505] Marion L (2018) Viktigt att bemöta alternativa fakta. Läkartidningen, 13 February. Available at: https://lakartidningen.se/klinik-och-vetenskap-1/reflexion/2018/02/viktigt-att-bemota-alternativa-fakta/

[bibr32-09636625221107505] Maslen S Lupton D (2019) ‘Keeping It Real’: Women’s enactments of lay health knowledges and expertise on Facebook. Sociology of Health & Illness 41(8): 1637–1651. 31392762 10.1111/1467-9566.12982

[bibr33-09636625221107505] Nguyen BT Allen AJ (2018) Social media and the intrauterine device: A YouTube content analysis. BMJ Sexual & Reproductive Health 44(1): 28–32. 29170151 10.1136/bmjsrh-2017-101799

[bibr34-09636625221107505] Nisbet MC Scheufele DA (2009) What’s next for science communication? Promising directions and lingering distractions. American Journal of Botany 96(10): 1767–1778. 21622297 10.3732/ajb.0900041

[bibr35-09636625221107505] Rabeharisoa V Moreira T Akrich M (2014) Evidence-based activism: Patients’, users’ and activists’ groups in knowledge society. Biosocieties 9: 111–128.

[bibr36-09636625221107505] Rentschler C (2014) Rape culture and the feminist politics of social media. Girlhood Studies 7(1): 65–82.

[bibr37-09636625221107505] Seidel S Kreutzer R Smith D McNeel S Gilliss D (2001) Assessment of commercial laboratories performing hair mineral analysis. JAMA 285(1): 67–72. 11150111 10.1001/jama.285.1.67

[bibr38-09636625221107505] Shackley S Wynne B (1996) Representing uncertainty in global climate change science and policy: Boundary-ordering devices and authority. Science, Technology, & Human Values 21(3): 275–302.

[bibr39-09636625221107505] Shaw I (2002) How lay are lay beliefs? Health 6(3): 287–299.

[bibr40-09636625221107505] Smith N Graham T (2019) Mapping the anti-vaccination movement on Facebook. Information, Communication & Society 22(9): 1310–1327.

[bibr41-09636625221107505] Smith S Pandit A Rush S (2015) The association between patient activation and accessing online health information: Results from a national survey of US adults. Health Expect 18: 3262–3273. 25475371 10.1111/hex.12316 PMC5810745

[bibr42-09636625221107505] Southwell BG Niederdeppe J Cappella JN Gaysynsky A Kelley DE Oh A , et al. (2019) Misinformation as a misunderstood challenge to public health. American Journal of Preventive Medicine 57(2): 282–285. 31248741 10.1016/j.amepre.2019.03.009

[bibr43-09636625221107505] Swedish Medical Products Agency (2018) Rapport Tillsynsärende 6.6.3-2017-18405. Uppsala: Swedish Medical Products Agency.

[bibr44-09636625221107505] Tomes N Hoffman B (2011) Introduction: Patients as policy actors. In: Hoffman B Tomes N Grob R Schlesinger M (eds) Patients as Policy Actors. New Brunswick, NJ: Rutgers University Press, pp. 1–16.

[bibr45-09636625221107505] Vuolanto P Bergroth H Nurmi J Salmenniemi S (2020) Reconfiguring health knowledges? Contemporary modes of self-care as ‘everyday fringe medicine’. Public Understanding of Science 29(5): 508–523. 32597366 10.1177/0963662520934752 PMC7411526

[bibr46-09636625221107505] Wang Y McKee M Torbica A Stuckler D (2019) Systematic literature review on the spread of health-related misinformation on social media. Social Science & Medicine 240: 112552. 31561111 10.1016/j.socscimed.2019.112552 PMC7117034

[bibr47-09636625221107505] Wemrell M Gunnarsson L (2022a) Attitudes toward HPV vaccination in Sweden: A survey study. Frontiers in Public Health 10: 729497. 35619814 10.3389/fpubh.2022.729497 PMC9127737

[bibr48-09636625221107505] Wemrell M Gunnarsson L (2022b) Attitudes towards the copper IUD among women in Sweden: a survey study. Frontiers in Global Women’s Health. DOI: 10.3389/fgwh.2022.920298 10.3389/fgwh.2022.920298 PMC9304811 35873134

[bibr49-09636625221107505] Wynne B (2006) Public engagement as a means of restoring public trust in science – hitting the notes, but missing the music? Public Health Genomics 9(3): 211–220. 10.1159/000092659 16741352

[bibr50-09636625221107505] Xu ZL (2005) Science and scientificity. Genomics, Proteomics & Bioinformatics 3(4): 197–200. 10.1016/S1672-0229(05)03026-3 PMC5172557 16689685

[bibr51-09636625221107505] Yunus MB (2001) The role of gender in fibromyalgia syndrome. Current Rheumatology Reports 3(2): 128–134. 11286669 10.1007/s11926-001-0008-3

[bibr52-09636625221107505] Zavestoski S Morello-Frosch R Brown P Mayer B McCormick S Altman RG (2004) Embodied health movements and challenges to the dominant epidemiological paradigm. In: Cress DM Myers DJ (eds) Authority in Contention. Bingley: Emerald, pp. 253–278.

