# Supplementary material for: On the verge between the scientific and the alternative: Swedish women’s claims about systemic side effects of the copper intrauterine device
Source: Public Underst Sci. 2022 Jul 28;32(2):175–89. doi: 10.1177/09636625221107505 (PMC9900186; doi:10.1177/09636625221107505)
Supplement: sj-docx-1-pus-10.1177_09636625221107505 – Supplemental material for On the verge between the scientific and the alternative: Swedish women’s claims about systemic side effects of the copper intrauterine device [file sj-docx-1-pus-10.1177_09636625221107505.docx]

On the verge between the scientific and the alternative: Swedish women’s claims about systemic side effects of the copper IUD

Supplemental material: Interview guide

Lena Gunnarsson and Maria Wemrell

School of Humanities, Education and Social Sciences, Örebro University, Örebro, Sweden

Department of Gender Studies, Lund University, Lund, Sweden, and Unit for Social Epidemiology, Department of Clinical Sciences, Lund University, Malmö, Sweden

Correspondence to: Lena Gunnarsson, School of Humanities, Education and Social Sciences, Örebro University, 701 82 Örebro, Sweden, lena.gunnarsson@oru.se

**Interview Guide**

- Introduction and consent procedure
- How did you come to suspect or be convinced that using a copper IUD may lead to side effects that are not recognized by established healthcare providers?
- How do you navigate among information and claims about the copper IUD?
- In case of personal experiences of suspected symptoms: What is your experience of seeking help from healthcare regarding this issue?
- How do you think that healthcare staff should relate to women who claim to suffer from unestablished side effects of the copper IUD?
- What has the ‘copper Facebook group’ meant to you?
- Follow-up questions on interesting/relevant themes throughout the interviews
